# Complete sequence of carbapenem-resistant *Ralstonia mannitolilytica* clinical isolate co-producing novel class D β-lactamase OXA-1176 and OXA-1177 in Japan

**DOI:** 10.1128/spectrum.03919-23

**Published:** 2024-03-14

**Authors:** Wataru Hayashi, Hiroyuki Kaiju, Shizuo Kayama, Liansheng Yu, Hui Zuo, Yo Sugawara, Kaoru Azuma, Akemi Takahashi, Yuka Hata, Motoyuki Sugai

**Affiliations:** 1 Antimicrobial Resistance Research Center, National Institute of Infectious Diseases, Tokyo, Japan; 2 Department of Laboratory Medicine, Mie Prefectural General Medical Center, Mie, Japan; University at Albany, Albany, New York, USA

**Keywords:** *Ralstonia mannitolilytica*, carbapenemase, OXA-1176, OXA-1177

## Abstract

In 2020, the *Ralstonia mannitolilytica* strain JARB-RN-0044 was isolated from a midstream urine sample of an elderly hospitalized patient in Japan and was highly resistant to carbapenem (i.e., imipenem, meropenem, and doripenem). Whole-genome sequencing revealed that the complete genome consists of two replicons, a 3.5-Mb chromosome and a 1.5-Mb large non-chromosomal replicon which has not been reported in *R. mannitolilytica,* and referred to as the “megaplasmid” in this study based on Cluster of Orthologous Group of proteins functional analysis. The strain JARB-RN-0044 harbored two novel OXA-60 and OXA-22 family class D β-lactamase genes *bla*_OXA-1176_ and *bla*_OXA-1177_ on the megaplasmid. Cloning experiments indicated that *Escherichia coli* recombinant clone expressing *bla*_OXA-1176_ gene showed increased minimum inhibitory concentrations (MICs) of imipenem, meropenem, and doripenem, indicating that *bla*_OXA-1176_ gene encodes carbapenemase. In contrast, *E. coli* recombinant clone expressing *bla*_OXA-1177_ gene showed increased MICs of piperacillin and cefazolin, but not of carbapenem. Interestingly, the 44.6 kb putative prophage region containing genes encoding phage integrase, terminase, head and tail protein was identified in the downstream region of *bla*_OXA-1176_ gene, and comparative analysis with some previously reported *R. mannitolilytica* isolates revealed that the prophage region was unique to strain JARB-RN-0044. The existence of a highly carbapenem-resistant *R. mannitolilytica* isolate may raise human health concerns in Japan, where the population is rapidly aging.

**IMPORTANCE:**

*Ralstonia mannitolilytica* is an aerobic non-fermenting Gram-negative rod commonly found in aquatic environments and soil. The bacteria can occasionally cause severe hospital-acquired bloodstream infections in immunocompromised patients and it has been recently recognized as an emerging opportunistic human pathogen. Furthermore, some *R. mannitolilytica* isolates are resistant to various antimicrobial agents, including β-lactams and aminoglycosides, making antimicrobial therapy challenging and clinically problematic. However, clinical awareness of this pathogen is limited. To our knowledge, in Japan, there has been only one report of a carbapenem-resistant *R. mannitolilytica* clinical isolate from urine by Suzuki et al. in 2015. In this study, whole-genome sequencing analysis revealed the presence and genetic context of novel *bla*_OXA-1176_ and *bla*_OXA-1177_ genes on the 1.5 Mb megaplasmid from highly carbapenem-resistant *R. mannitolilytica* isolate and characterized the overall distribution of functional genes in the chromosome and megaplasmid. Our findings highlight the importance of further attention to *R. mannitolilytica* isolate in clinical settings.

## OBSERVATION

*Ralstonia mannitolilytica* is an aerobic non-fermenting Gram-negative rod commonly found in aquatic environments and soil (1). The bacteria can occasionally cause severe hospital-acquired bloodstream infections in immunocompromised patients (2–5). The disease caused by *R. mannitolilytica* can progress rapidly and sometimes lead to septic shock symptoms and multiple organ dysfunction syndromes (1, 6). However, clinical awareness of this rare pathogen is limited. In addition, some *R. mannitolilytica* isolates are resistant to various antimicrobial agents, including β-lactams and aminoglycosides, making antimicrobial therapy challenging and clinically problematic (4, 5). Recently, healthcare-associated infections due to *R. mannitolilytica* related to contaminated water and medical devices have been reported worldwide (7–9), and it is increasingly recognized as an emerging opportunistic human pathogen.

In 2020, the *R. mannitolilytica* strain JARB-RN-0044 was isolated from a midstream urine sample of a female patient in her 70s in a general hospital in Mie Prefecture, southwest of the Chubu region, Japan. Antimicrobial susceptibility testing was performed with Neg MIC EN 2J (Beckman Coulter, Brea, CA, USA) and NQJ2 (Eiken Chemical Co., Tokyo, Japan) panels and interpreted according to minimum inhibitory concentration (MIC) breakpoints for *Pseudomonas aeruginosa* from Clinical and Laboratory Standards Institute documents M100-ED30 guidelines (10). The isolate exhibited high MICs against piperacillin, imipenem, meropenem, and doripenem; however, it remained susceptible to levofloxacin and ciprofloxacin (Table S1). The isolate was negative for screenings of carbapenemase producers using modified carbapenem inactivation methods, mCIM and CIMTris (11, 12).

The isolate was subjected to short-read and long-read whole-genome sequencing using Hiseq X Five (Illumina Inc., San Diego, CA, USA) and GridION (Oxford Nanopore Technologies, Oxford, UK) to understand the resistance mechanism. The hybrid assembly of both reads was conducted using Unicycler v0.4.8 pipeline (13). Subsequently, genome annotation was performed by using the DDBJ Fast Annotation and Submission Tool (DFAST) v1.2.16 (14), and functional genes were assigned to Cluster of Orthologous Group of proteins (COG) categories using eggNOG-mapper v2.1.9 (15). Antimicrobial resistance genes were identified by using ABRicate v1.0 (https://github.com/tseemann/abricate) with the CARD database (16). The PHASTER webserver was used to predict prophage sequences (17).

**Fig 1:**
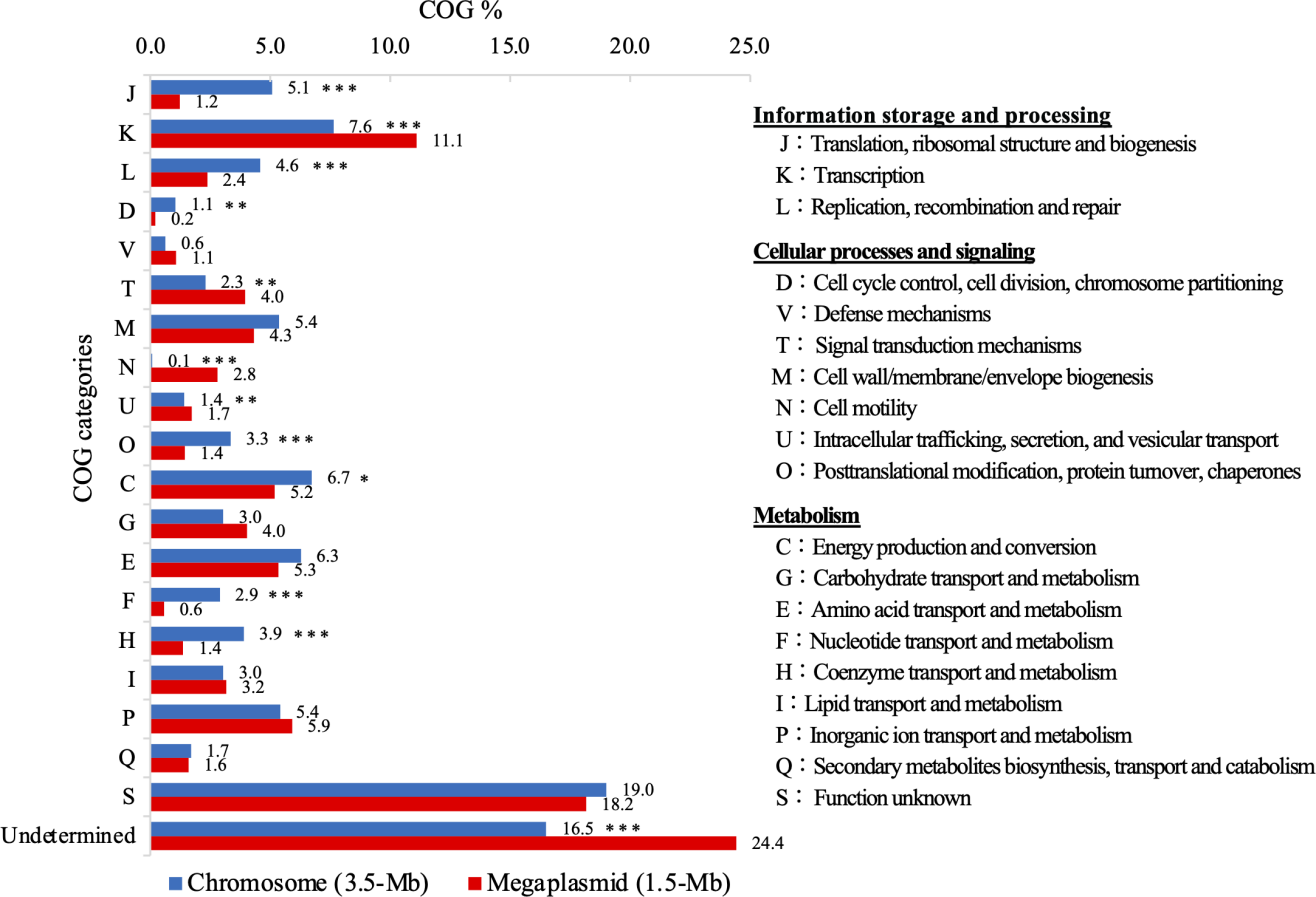
Distribution of COG categories between the chromosome and megaplasmid harbored by the *R. mannitolilytica* strain JARB-RN-0044. Asterisks indicate statistically significant differences (*P < 0.05, **P < 0.01, and ***P < 0.001 by Fisher’s exact test).

The *R. mannitolilytica* strain JARB-RN-0044 was identified by using matrix-assisted laser desorption ionization-time of flight mass spectrometry (Bruker Daltonics Japan, Yokohama, Japan) with a score of 2.38. Pair-wise average nucleotide identity based on MUMmer calculation (ANIm) analysis using JSpeciesWS online tool (18) revealed that strain had the highest ANIm value of 99.4% with the *R. mannitolilytica* strain WCHRM065837 (GenBank assembly accession number GCA_002939145). The hybrid assembly yielded a large circular replicon with a size of 1.5 Mb in addition to a chromosome with a size of 3.5 Mb with a mean G + C content of 66.1% (Fig. S1). To date, the functional role of large non-chromosomal replicon as the “megaplasmid” in other *Ralstonia* spp. has been clarified, while it has been completely unknown in *R. mannitolilytica*. The 3.5 Mb chromosome harbored by strain JARB-RN-0044 carries several localized housekeeping genes (*rpoB*, *rnpA*, *rpmH*, *dnaA*, *dnaN*, and *gyrB*) and two copies each of the 16S, 23S, and 5S rRNA genes; however, no rRNA genes were found on the 1.5 Mb replicon. The 1.5 Mb replicon carried the *repA-parA-parB* gene cluster involved in plasmid replication and partitioning systems and 12 conserved iteron repeats (G/CCGTACCCG/ATTTCTGCG) essential for RepA binding previously identified in *Ralstonia solanacearum* isolate (18). Overall, the 3.5 Mb chromosome and 1.5 Mb replicon consisted of 3,336 and 1,390 protein-coding genes, respectively, and the COG were mainly classified into 19 categories, more than half of which had different distribution characteristics between the two replicons (Fig. 1). Particularly, gene families involved in replication, recombination, repair, and metabolism of nucleotides and coenzymes were distributed primarily on the chromosome compared with the 1.5 Mb replicon (Fisher’s exact test, *P*  < 0.001); however, those involved in transcription and cell motility were significantly abundant on the 1.5 Mb replicon (*P*  < 0.001). As this was similar to a previously described functional distribution found in megaplasmids possessed by *R. solanacearum* strains (19–22), we refer to the 1.5 Mb replicon as a presumptive “megaplasmid” in this study.

The *R. mannitolilytica* strain JARB-RN-0044 harbored two novel OXA-60 and OXA-22 family class D β-lactamase genes encoding carbapenemase and narrow spectrum oxacillinase on the megaplasmid, which were newly assigned as *bla*_OXA-1176_ (RefSeq accession number NG_148663) and *bla*_OXA-1177_ (RefSeq accession number NG_148664) genes, respectively. OXA-1176 and OXA-1177 had two amino acid substitutions compared with OXA-571 (GenBank accession number MG736318) and OXA-572 (GenBank accession number MG736319) possessed by the *R. mannitolilytica* isolate WCHRM065837 in China, respectively, and both shared 99.3% amino acid identity each. The amplified products of *bla*_OXA-1176_ and *bla*_OXA-1177_ genes were subcloned into the pTAKN-2 vector (BioDynamics Laboratory Inc., Tokyo, Japan) carrying kanamycin resistance gene and chemically transformed into ECOS X Competent *Escherichia coli* DH5α (Nippon Gene, Tokyo, Japan). The cloning and expression of *bla*_OXA-1176_ gene clearly indicated that the *E. coli* DH5α recombinant clone harboring pTAKN-2/OXA-1176 showed more than fourfold increases in the MICs of imipenem, meropenem, and doripenem, indicating that OXA-1176 is presumed to have hydrolytic activity against carbapenem, consistent with previous report (23) (Table S1). In contrast, the *E. coli* DH5α recombinant clone harboring pTAKN-2/OXA-1177 was susceptible to all carbapenems tested; however, it showed a 16-fold increase in the MICs of piperacillin and cefazolin, but not of carbapenem.

**Fig 2:**
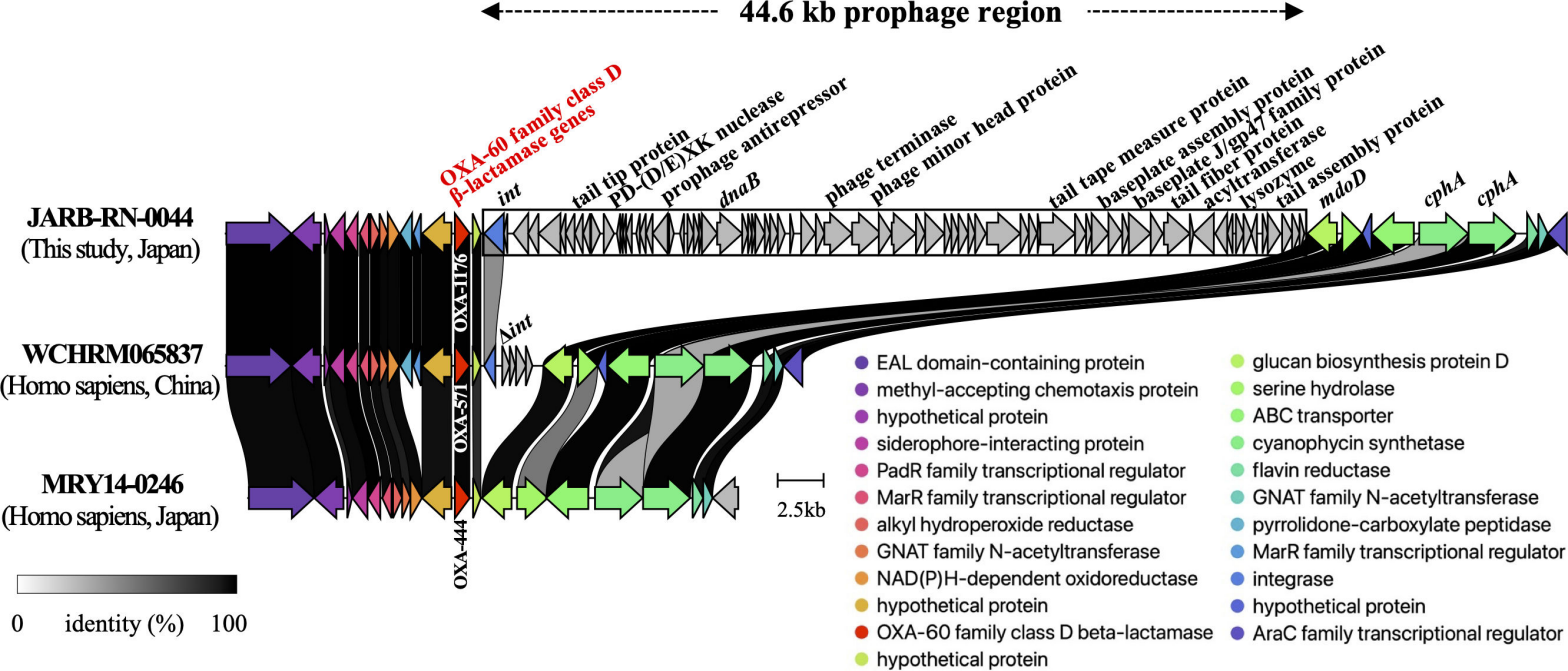
Linear comparison of the genetic environment surrounding the *bla*_OXA-1176_ gene in the *R. mannitolilytica* strain JARB-RN-0044 (this study), including ca. the 44.6 kb prophage-related sequence (surrounded by a square) with the genetic environment of OXA-60 family class D β-lactamase genes *bla*_OXA-571_ and *bla*_OXA-444_ harbored by the *R. mannitolilytica* strain WCHRM065837 (GenBank assembly accession number GCA_000953875.1) and MRY14-0246 (GenBank assembly accession number GCA_000953875.1), respectively. The arrows, marked with a specific color, show the direction of the predicted coding genes. This figure was generated using clinker v0.0.25 (https://github.com/gamcil/clinker).

Notably, the ca. 44.6 kb putative prophage region containing genes encoding phage integrase, terminase, head and tail protein was identified in the downstream region of *bla*_OXA-1176_ gene (Fig. 2). Attachment sites *attL* and *attR* were not detected at the ends of this prophage region. Comparative analysis with previously reported *R. mannitolilytica* isolates WCHRM065837 and MRY14-0246 (GenBank assembly accession number GCA_000953875.1) harboring OXA-60 family class D β-lactamase genes *bla*_OXA-571_ and *bla*_OXA-444_, respectively, revealed that the upstream regions of these genes were conserved, while the downstream regions were relatively variable, and the prophage region was unique to strain JARB-RN-0044, even though the disrupted *int* gene was found in strain WCHRM065837. BLAST searches did not show any homologous sequences to this prophage region; thus, its origin could not be inferred, and future studies to elucidate the function of this prophage and its association with the *bla*_OXA-1176_ gene are warranted. Meanwhile, *bla*_OXA-1177_ gene was flanked upstream by tRNA^Leu^ gene and downstream by the genes encoding the ArsR family transcriptional regulator and major facilitator superfamily (MFS) transporter, both of which were also found in strain WCHRM065837 harboring *bla*_OXA-572_ gene, and not in strain MRY14-0246 harboring *bla*_OXA-443_ gene (Fig. S2).

In conclusion, this study confirmed the presence and genetic context of novel *bla*_OXA-1176_ and *bla*_OXA-1177_ genes on the 1.5 Mb megaplasmid and the overall distribution of functional genes. However, there were several essential genes on the megaplasmid that may be crucial for bacterial survival, such as amino acid and nucleotide metabolic systems, as well as on the chromosome, and further studies are required to better understand the role of this large replicon. To the best of our knowledge, in Japan, there has been only one report of a carbapenem-resistant *R. mannitolilytica* strain from urine in 2015, as described above (24). The current trends of this opportunistic pathogen in Japan, which has limited treatment options, remain elusive. A recent study has also reported bloodstream infection caused by *R. mannitolilytica* isolate in a hospitalized patient with coronavirus disease 2019 (COVID-19) and the higher abundance of *Ralstonia* spp. in the gut microbiota of patients with severe COVID-19 (25, 26). The existence of a highly carbapenem-resistant *R. mannitolilytica* isolate may raise human health concerns in Japan, where the population is rapidly aging and requires further attention to this rare pathogen in clinical settings.

## Data Availability

This whole-genome sequencing project for the *R. mannitolilytica* strain JARB-RN-0044 has been deposited in the DDBJ/EMBL/GenBank under BioProject accession number PRJDB14781 and BioSample accession number SAMD00557611. The DDBJ Sequence Read Archive number is DRA015160. The nucleotide sequences of two novel class D β-Lactamase genes *bla*_OXA-1176_ and *bla*_OXA-1177_ reported in this study have been deposited in the RefSeq database under accession numbers NG_148663 and NG_148664, respectively.

## SUPPLEMENTAL MATERIAL

Fig. S1 spectrum.03919-23-s0001.pdf Circular representation of the chromosome and megaplasmid harbored by the *R. mannitolilytica* strain JARB-RN-0044.

Fig. S2 spectrum.03919-23-s0002.pdf Linear comparison of genetic environment surrounding the *bla*_OXA-1177_ gene in the *R. mannitolilytica* strain JARB-RN-0044 with the genetic environment of OXA-22 family class D β-lactamase genes *bla*_OXA-572_ and *bla*_OXA-443_ harbored by the *R. mannitolilytica* strains WCHRM065837 and MRY14-0246, respectively.

Table S1 spectrum.03919-23-s0003.pdf MICs of antimicrobial agents for the *R. mannitolilytica* strain JARB-RN-0044 and *E. coli* DH5α transformants expressing OXA-1176 and OXA-1177.

ASM does not own the copyrights to Supplemental Material that may be linked to, or accessed through, an article. The authors have granted ASM a non-exclusive, world-wide license to publish the Supplemental Material files. Please contact the corresponding author directly for reuse.
