# Supplementary material for: Complete sequence of carbapenem-resistant Ralstonia mannitolilytica clinical isolate co-producing novel class D β-lactamase OXA-1176 and OXA-1177 in Japan
Source: Microbiol Spectr. 2024 Mar 14;12(4):e03919-23. doi: 10.1128/spectrum.03919-23 (PMC10986519; doi:10.1128/spectrum.03919-23)
Supplement: Fig. S1 — Circular representation of the chromosome and megaplasmid harbored by the R. mannitolilytica strain JARB-RN-0044. [file spectrum.03919-23-s0001.pdf]

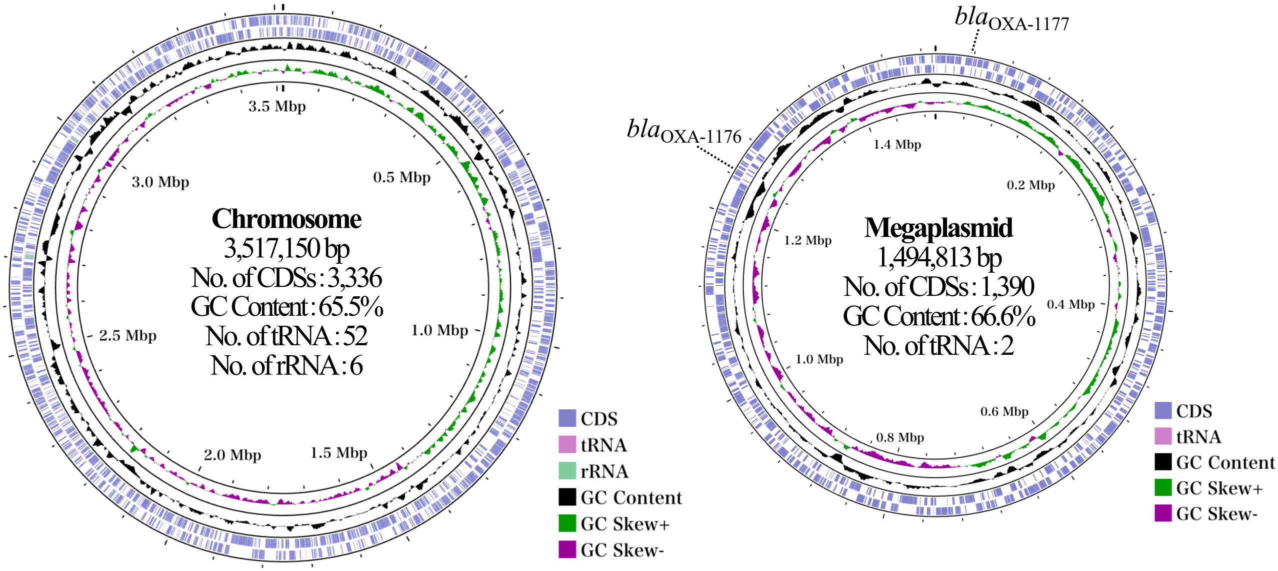

Fig. S1. Circular representation of the chromosome and megaplasmid harbored by the *R. mannitolilytica* strain JARB-RN-0044. The predicted coding genes, RNA (tRNA and rRNA), GC content and GC skew are depicted. This figure was generated using CGView server (https://cgview.ca).
