# Supplementary material for: Complete sequence of carbapenem-resistant Ralstonia mannitolilytica clinical isolate co-producing novel class D β-lactamase OXA-1176 and OXA-1177 in Japan
Source: Microbiol Spectr. 2024 Mar 14;12(4):e03919-23. doi: 10.1128/spectrum.03919-23 (PMC10986519; doi:10.1128/spectrum.03919-23)
Supplement: Fig. S2 — Linear comparison of genetic environment surrounding the blaOXA-1177 gene in the R. mannitolilytica strain JARB-RN-0044 with the genetic environment of OXA-22 family class D β-lactamase genes blaOXA-572 and blaOXA-443 harbored by the R. mannitolilytica strains WCHRM065837 and MRY14-0246, respectively. [file spectrum.03919-23-s0002.pdf]

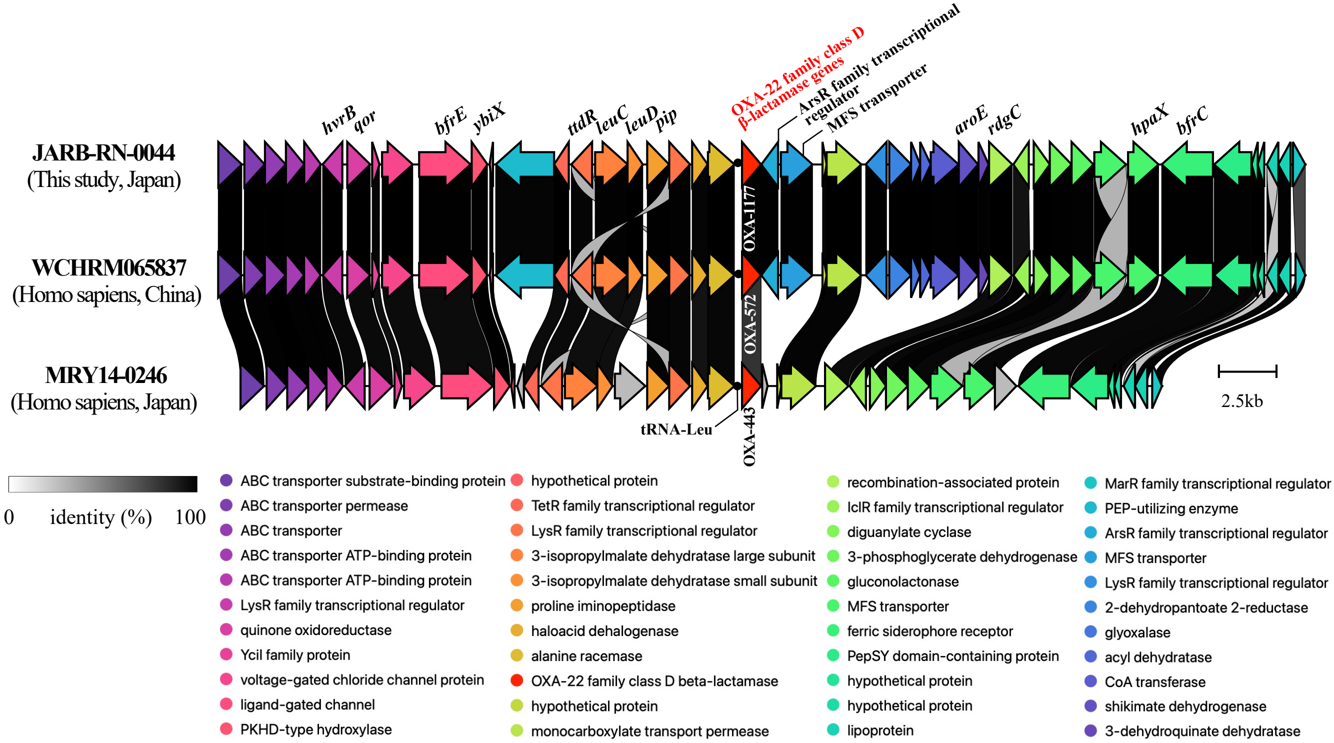

Fig. S2. Linear comparison of genetic environment surrounding the *bla*$_{OXA-1177}$ gene in the *R. mannitolilytica* strain JARB-RN-0044 (this study) with the genetic environment of OXA-22 family class D β-lactamase genes *bla*$_{OXA-572}$ and *bla*$_{OXA-443}$ harbored by the *R. mannitolilytica* strain WCHRM065837 and MRY14-0246, respectively.
