# Supplementary material for: Complete sequence of carbapenem-resistant Ralstonia mannitolilytica clinical isolate co-producing novel class D β-lactamase OXA-1176 and OXA-1177 in Japan
Source: Microbiol Spectr. 2024 Mar 14;12(4):e03919-23. doi: 10.1128/spectrum.03919-23 (PMC10986519; doi:10.1128/spectrum.03919-23)
Supplement: Table S1 — MICs of antimicrobial agents for the R. mannitolilytica strain JARB-RN-0044 and E. coli DH5α transformants expressing OXA-1176 and OXA-1177. [file spectrum.03919-23-s0003.pdf]

Table S1 Minimum inhibitory concentrations (MICs) for antimicrobial agents for the *R. mannitolilytica* strain JARB-RN-0044 and *E. coli* DH5α transformants expressing OXA-1176 and OXA-1177.

Antimicrobial agents	MICs (mg/L)			
	R. mannitolilytica JARB-RN-0044	*E. coli* DH5α transformant (pTAKN-2/OXA-1176)	*E. coli* DH5α transformant (pTAKN-2/OXA-1177)	*E. coli* DH5α (pTAKN-2)
Ampicillin	>16	>16	>16	≤4
Ampicillin/Sulbactam	>16/8	>16/8	≤4/2	≤4/2
Piperacillin	>64	8	>64	≤4
Piperacillin/Tazobactam	≤4/4	≤4/4	≤4/4	≤4/4
Amoxicillin/Clavulanic acid	>16/8	>16/8	≤8/4	≤8/4
Cefoperazone/Sulbactam	≤8/4	≤8/4	≤8/4	≤8/4
Cefazolin	>16	2	>16	≤1
Cefotiam	>16	≤8	≤8	≤8
Cefaclor	>16	≤8	16	≤8
Cefpodoxime	>4	≤1	≤1	≤1
Cefditoren	>2	≤1	≤1	≤1
Cefotaxime	>2	≤0.5	≤0.5	≤0.5
Ceftazidime	>8	≤1	≤1	≤1
Ceftriaxone	>2	≤0.5	≤0.5	≤0.5
Cefepime	>16	≤1	≤1	≤1
Cefmetazole	>32	≤4	≤4	≤4
Flomoxef	>32	≤8	≤8	≤8
Aztreonam	>8	≤1	≤1	≤1
Faropenem	16	4	2	1
Ertapenem	>1	≤0.25	≤0.25	≤0.25
Biapenem	64	2	≤0.06	≤0.06
Tebipenem	64	1	≤0.06	≤0.06
Imipenem	32	1	0.25	0.25
Meropenem	128	0.5	≤0.06	≤0.06

Doripenem	>64	1	≤0.06	≤0.06
Gentamicin	>8	≤2	≤2	≤2
Amikacin	>32	≤8	≤8	≤8
Minocycline	≤2	≤2	≤2	≤2
Levofloxacin	1	≤0.12	≤0.12	≤0.12
Ciprofloxacin	≤0.5	≤0.5	≤0.5	≤0.5
Fosfomycin	>16	≤4	≤4	≤4
Sulfamethoxazole–Trimethoprim	≤2/38	≤2/38	≤2/38	≤2/38
Tigecycline	≤0.5	≤0.5	≤0.5	≤0.5
